# The Impact of Lipid Digestion on the Dynamic and Structural Properties of Micelles

**DOI:** 10.1002/smll.202004761

**Published:** 2021-01-20

**Authors:** Demi L. Pink, Fabrizia Foglia, David J. Barlow, M. Jayne Lawrence, Christian D. Lorenz

**Affiliations:** ^1^ Department of Physics King's College London London WC2R 2LS UK; ^2^ Department of Chemistry University College London 20 Gordon Street London WC1H 0AJ UK; ^3^ Faculty of Life Sciences and Medicine King's College London Franklin‐Wilkins Building, Stamford Street London SE1 9NH UK; ^4^ Division of Pharmacy and Optometry School of Health Sciences Faculty of Biology, Medicine and Health University of Manchester Stopford Building, Oxford Road Manchester M13 9PL UK

**Keywords:** drug delivery vehicles, lipid digestion, lipid micelles, molecular dynamics simulations, phosphocholine lipids, small‐angle neutron scattering

## Abstract

Self‐assembled, lipid‐based micelles, such as those formed by the short‐chain phosphocholine, dihexanoylphosphatidylcholine (2C6PC), are degraded by the pancreatic enzyme, phospholipase A_2_ (PLA2). Degradation yields 1‐hexanoyl‐lysophosphocholine (C6LYSO) and hexanoic acid (C6FA) products. However, little is known about the behavior of these products during and after the degradation of 2C6PC. In this work, a combination of static and time‐resolved small angle neutron scattering, as well as all‐atom molecular dynamics simulations, is used to characterize the structure of 2C6PC micelles. In doing so a detailed understanding of the substrate and product aggregation behavior before, during and after degradation is gained. Consequently, the formation of mixed micelles containing 2C6PC, C6LYSO and C6FA is shown at every stage of the degradation process, as well as the formation of mixed C6LYSO/C6FA micelles after degradation is complete. The use of atomistic molecular dynamics has allowed us to characterize the structure of 2C6PC, 2C6PC/C6LYSO/C6FA, and C6LYSO/C6FA micelles throughout the degradation process, showing the localization of the different molecular species within the aggregates. In addition, the hydration of the 2C6PC, C6LYSO, and C6FA species both during micellization and as monomers in aqueous solution is documented to reveal the processes driving their micellization.

## Graphical abstract

This work uses molecular dynamics simulations and small‐angle neutron scattering to study the self‐assembly and degradation of micelles formed by the short‐chain phosphocholine lipid, 1,2‐dihexanoyl‐sn‐glycero‐3‐phosphocholine. The internal structure of the micelles formed before, during and after degradation by the PLA2 enzyme is detailed and changes in hydration for all species throughout the process are documented.

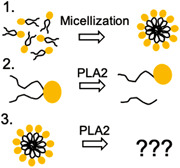

## 1 Introduction

In the past few decades, there has been an increasing number of nanoparticle formulations fabricated; many of which have been investigated for a range of biomedical applications, including the delivery of hydrophobic drugs. Among the nanoparticles produced, those composed of lipids, that is, lipid nanoparticles, have been of particular importance due to their biocompatibility.^[^ ^1^, ^2^, ^3^ ^]^ One class of lipid that is of great interest is phosphatidylcholine (PC).

PC molecules are biologically important as they form the major components of cell membranes. Like many other lipids they are amphiphilic and, depending upon their molecular structure, can be tailored to self‐assemble into micelles, bilayers or other nanostructures. For example, small alterations in the length of the lipid tails can dramatically change both the concentration at which the lipids self‐assemble, known as the critical micelle concentration (CMC), and the nature of the aggregates they form. As the length of the hydrocarbon tails increases so does the size of the resulting aggregates.^[^ ^4^ ^]^ Longer chain lipids (more than 14 carbons per tail) typically form bilayers at a lower CMC whilst the shorter chain lipids (less than eight carbons per tail) form micelles at a higher CMC.^[^ ^4^ ^]^

The aggregates formed by PC molecules have been investigated using a combination of experimental and computational techniques. These techniques include nuclear magnetic resonance,^[^ ^5^ ^]^ specular neutron reflection,^[^ ^6^ ^]^ and atomistic and coarse‐grained MD simulations.^[^ ^7^ ^]^ However, studies have typically focused on the self‐assembly and degradation of the liposomes and bilayers formed by long‐chain (as opposed to short‐chain) PC molecules. Within the body, PC molecules are degraded via hydrolysis of the PC sn‐2 ester bond, mediated by the calcium‐dependent phospholipase A_2_ (PLA2) enzyme.^[^ ^8^ ^]^ This results in the formation of a lysophosphocholine (LYSO) and fatty acid (FA).

Although there have been numerous studies into the kinetics of PLA2 hydrolysis at bi‐layers and, to a lesser extent, micelles, there has been little examination of the internal structure of the resulting aggregates. An investigation into the degradation, using PLA2, of dipalmitoylphosphatidylcholine (DOPC) membranes in the form of bilayers or vesicles found that the LYSO and FA products accumulated at the surface of the membrane due to their poor transport within the solution.^[^ ^9^, ^10^ ^]^ However, after washing of the lipid membrane with buffer, the lysoPC and FA desorbed from the surface.^[^ ^9^ ^]^ In another study, the hydrolysis of cubic micellar structures composed of equal amounts of soy PC and glycerol dioleate (GDO) was investigated, in which the bilayer layer forming soy PC was degraded into a reversed micellar lipid phase.^[^ ^11^ ^]^ However, all of these studies involved the use of long‐chain lipids (>14 carbons). As indeed did the investigation into the products of dipalmitoylphosphatidylcholine (DPPC) degradation which found that solutions containing either only LYSO or only FA molecules both formed micelles, whilst mixtures of LYSO/FA formed bilayers.^[^ ^11^ ^]^

PLA2 is considerably more active toward phospholipids that form part of a micelle as compared to when they are in the form of a bilayer or vesicle.^[^ ^8^ ^]^ This difference is thought to be a consequence of the PC head group occupying a larger surface area when in a prolate ellipsoidal micelle as opposed to a bilayer (i.e., 1.02 nm^2^ compared with 0.65 nm^2^).^[^ ^12^ ^]^ The increased head group area leads to increased adsorption and therefore activity of PLA2.^[^ ^13^ ^]^ Short chain PC lipids spontaneously self‐assemble into micelles rather than lamellar structures. The micelles formed by short chain PC lipids have other advantageous properties including a monodispersity of size and shape and generally small micelle sizes. However, there has been little investigation of the fate of the LYSO and FA products during and after the degradation of short‐chain PC aggregates. As a result, we have focused our investigation on the self‐assembly and degradation of short‐chain PC micelles as opposed to vesicles or similar structures.

Many of the studies conducted, on both long and short chain PC aggregates, use natural phospholipids. Phospholipids from natural sources, such as egg phosphocholine and soy phosphocholine, can differ in their exact composition and contain fatty acid chains of varying length and saturation. Despite this, natural phospholipids are often preferred in pharmaceutical formulations over synthetic phospholipids where there is a need for their large‐scale production.^[^ ^14^ ^]^

It is particularly challenging to derive insight into the fate of a particular PC species when it forms part of a mixture as the presence of additional components in the membranes such as other lipid types, fatty acids and cholesterol can greatly impact the arrangement and dynamics of the PC products such as the formation of micelles compared to bilayers.^[^ ^15^ ^]^ In this study, we have elected to study the aggregates formed from a single PC lipid, 1,2‐dihexanoyl‐sn‐glycero‐3‐phosphocholine (2C6PC).

To determine the fate of the C6LYSO and C6FA products, this work has used atomistic simulations to model the self‐assembly of the initial 2C6PC micelle, followed by modeling the 2C6PC lipids alongside C6FA and C6LYSO in different ratios of parent:product molecules. This allows us to model the states of degradation of the micelle over time. This simulation work is complemented by experimental data gathered using small‐angle neutron scattering (SANS). By combining simulation and experimental data we are able to gain a multi‐scale understanding of the fate of the 2C6PC lipid molecules and the products of their hydrolysis both during and after degradation.

## 2 Results and Discussion

### 2.1 Static Small Angle Neutron Scattering Measurements

**Figure 1:**
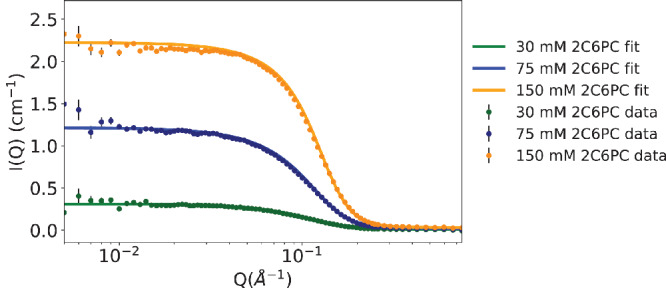
SANS data and model fits assuming a triaxial ‘lozenge’ shape for dispersions of varying 2C6PC concentrations in D_2_O containing TRIS buffer, 150 mm NaCl and 5 mm CaCl_2_ at a pD equivalent to a pH of 7.4. 150 mm 2C6PC, top, 75 mm 2C6PC center and 30 mm 2C6PC lower curves, respectively. I(Q) are scattering intensities, and Q is the neutron momentum transfer, in Å^−1^. Measurements were made at 298 ± 0.1 K. The structural parameters for the model fits shown are presented in **Table** **1**.

We have measured three concentrations of 2C6PC (30, 75 & 150 mm, which correspond to roughly 2×, 5× and 10× CMC, respectively) at 298 ± 0.1 K. Each concentration was investigated in pure D_2_O and two different D_2_O solutions containing TRIS buffer, NaCl (150 mm) and CaCl_2_ (5 M); one at a pD of 7, equivalent to a pH of 7.4, and the other at a pD of 7.6, equivalent to a pH of 8.0. We observed no difference in the SANS profiles of the same concentration of 2C6PC in the three different aqueous solvents tested (see Figure S6, Supporting Information). The lack of difference in the scattering profiles is expected due to the zwitterionic nature of the lipid and the relatively low level of added electrolyte. Furthermore, over the range of concentrations studied, the micelle size was not concentration dependent as was confirmed by calculating the *R*_g_ from the 30, 75, and 150 mm SANS profiles illustrated in **Figure** **1**. *R*_g_ values of 14.9 ± 0.01 Å, 14.9 ± 0.01 Å, and 14.2 ± 0.01 Å were obtained for the 30, 75, and 150 mm solutions respectively. The concentration independence of the size of 2C6PC micelles is in agreement with earlier studies.^[^ ^16^, ^17^ ^]^ It is worth noting, however, that the SANS data obtained for the weak scattering samples containing 30 mm of 2C6PC were very noisy due to the relatively high CMC (≈ 14 mm) recorded for 2C6PC^[^ ^4^, ^18^ ^]^ and the small size of the 2C6PC aggregates.^[^ ^16^, ^17^ ^]^

Whilst scattering experiments have been performed previously on the aggregates formed by 2C6PC, there have been conflicting interpretations of the aggregate's nature. For example, prior analysis of SANS studies of 2C6PC dispersed in aqueous solvent over the concentration range of 0.027 to 0.361 M suggested the formation of prolate core–shell ellipsoidal micelles^[^ ^16^ ^]^ with minor and major core radii of 7.8 and 24 Å, respectively, and a shell that varied slightly in thickness, being 10 and 6 Å in the direction of the minor and major axis, respectively. Previous static light scattering experiments investigating a narrower concentration range of 2C6PC with an upper limit of 0.044 M described the micelles as capped cylinders with a length (excluding ‘caps') of ≈ 48 Å and an outer radius (including core and shell) fixed at either 15 or 18 Å.^[^ ^17^ ^]^ Assuming either a prolate ellipsoid or a capped cylindrical micelle yielded aggregation numbers of 19 and 34 respectively.^[^ ^16^, ^17^ ^]^

In order to address the uncertainty in the literature, we fitted our SANS data using a number of different models, including core–shell spheroids (prolate, oblate and spherical), a core–shell capped cylinder and a core–shell triaxial micelle model. All models included a hard sphere structure factor to account for the presence of inter‐particulate interactions. The best fit to our SANS data (see Figure 1), regardless of solvent and 2C6PC concentration, was obtained when using a core–shell triaxial model, or more specifically a ‘lozenge’ shape (see Table 1 for parameters). Table 1 also shows some of the derived parameters including the aggregation number and number of water molecules per PC head group. While the use of other models to fit the SANS profiles often gave statistically comparable fits to the measured data they yielded geometries and/or lipid interfacial areas and/or head group hydrations that were physically unrealistic (not shown).

**Table 1:** Structural parameters used to obtain the best fit using a triaxial model of the SANS data recorded at 298 ± 0.1 K for 2C6PC micellar dispersions in D_2_O, containing TRIS buffer, 150 mm NaCl and 5 mm CaCl_2_ at a pD of 7.0, equivalent to a pH of 7.4. All radius values are given in Å. *R*_Minor_ and *R*_Major_ refer to the minor equatorial radius and the major equatorial radius respectively whilst *R*_Polar_ is used to describe the polar radius of the micelle. The aggregation number is labelled as N and H_2_O refers to the lipid head group hydration. The best fits to the SANS data are shown presented in Figure 1

2C6PC conc. [mm]	pH	Core *R*_Minor_	Core *R*_Major_	Core *R*_Polar_	*R*_Minor_	*R*_Major_	*R*_Polar_	N	H_2_O	Lipid area (core)	Lipid area (shell)
**30**	D_2_O	14.4 (±0.4)	45.6 (±4.2)	4.9 (±2.0)	19.4	37.3	9.9	29	12.2	115	199
**30**	7.4	15.1 (±0.4)	41.6 (±0.5)	4.4 (±2.2)	20.1	36.5	9.4	27	13.9	125	215
**30** ^a)^	8	15.7	42.9	6.4	20.7	32.1	11.4	35	8.3	93	161
**75**	D_2_O	14.9 (±0.1)	30.4 (±1.3)	6.1 (±1.3)	19.5	35.4	11.1	34	9.0	97	168
**75**	7.4	14.5 (±0.1)	29.7 (±0.8)	6.0 (±1.7)	19.9	34.7	11.0	34	9.1	98	169
**75**	8	15.7 (±0.4)	35.0 (±0.8)	5.8 (±1.6)	20.7	40.0	10.8	41	8.5	99	164
**150**	D_2_O	15.7 (±0.2)	27.1 (±0.3)	6.3 (±1.4)	20.7	32.1	11.3	35	8.0	91	158
**150**	7.4	16.0 (±0.1)	27.1 (±0.1)	6.2 (±3.6)	20.7	32.1	11.2	35	8.4	94	161
**150**	8	15.7 (±0.1)	27.1 (±0.3)	6.4 (±0.1)	21.0	32.1	11.4	34	8.7	95	165

^a)^ Parameter values for the 30 mm 2C6PC micelle solution could not be determined reliably by optimization owing to the poor statistics, and the values presented are those determined by manual fitting to the measured data; Note: symbols used are as described in the text, and the figures in parentheses show standard errors. Thickness of the shell region was fixed at 5 Å.

With the exception of the reduced quality results obtained for 30 mm (due to the lower 2C6PC concentration), the length of the polar radius was between 9.4 and 11.4 Å, that is, approximately the length of the 2C6PC molecule, in all three aqueous solvents tested. This value corresponds to the half ‘thickness’ of the lozenge and suggests that the arrangement of the 2C6PC molecules in the ‘lozenge’ approximate to a bilayer and that this bilayer extends for ≈20.5 Å × 34.5 Å. Furthermore, the size and shape of the 2C6PC aggregates at 75 and 150 mm concentrations were comparable. The aggregation number obtained, calculated using the volume of the hydrophobic core and the volume calculated for two C5 chains, was 35.5 ± 2.6 which is comparable to the aggregation number of 34 obtained from previous light scattering experiments.^[^ ^17^ ^]^ The area per 2C6PC at the boundary between the core and the head group region is twice the area generally found for longer alkyl chain PC lipids in a monolayer/bilayer whereas the thickness of the head group region was slightly smaller than measured for longer alkyl chain PC lipids. Taken together, these last two observations suggest that the phospholipid head group is lying relatively flat in the prolate ellipsoid micelle compared to the more extended conformation seen in the corresponding bilayer‐forming molecules. The number of water molecules per head group is 8.6 ± 0.4; this is toward the lower end of the range widely quoted for phosphatidylcholine head groups and is most likely the result of the flat conformation of the head group.

### 2.2 Time‐Resolved Small Angle Neutron Scattering Measurements

**Figure 2:**
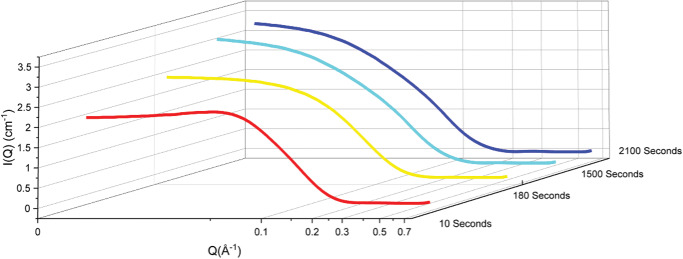
Best fit to the stopped‐flow SANS data at 298 ± 0.1 K using a triaxial (lozenge) shaped model for aqueous dispersions of containing an initial concentration of 150 mm of 2C6PC in TRIS buffer, 150 mm NaCl and 5 mm CaCl_2_ in protein matched water (PMW) at 7.4. *I*(*Q*) is the scattering intensity, and *Q* is the neutron momentum transfer, in Å ^−1^. The structural parameters for the model fits shown are presented in Table 1.

The degradation of the 75 and 150 mm solutions of 2C6PC at a constant ratio of PLA2 of 0.125 and 0.25 mg mL^−1^, respectively, was monitored using stopped flow SANS for a total of 75 and 55 min. The results of these experiments are summarized in **Table** **2**, and **Figure** **2** shows the changes in the scattering observed for the 150 mm 2C6PC solution.

**Table 2:** Structural parameters used to obtain the best fit using a triaxial model of the SANS data recorded at 298 ± 0.1 K for 2C6PC micellar dispersions in D_2_O, containing TRIS buffer, 150 mm NaCl and 5 mm CaCl_2_ at a pD equivalent to a pH of 7.4. All radial values are given in Å. *R*_Minor_ and *R*_Major_ refer to the minor equatorial radius and the major equatorial radius respectively whilst *R*_Polar_ is used to describe the polar radius of the micelle. The best fits to the SANS data are shown presented in Figure 2

150 mm 2C6PC + 0.25 mg mL^−1^ PLA				75 mm 2C6PC + 0.125 mg mL^−1^ PLA			
**Time [s]**	***R*_Minor_**	***R*_Major_**	***R*_Polar_**	**Time [s]**	***R*_Minor_**	***R*_Major_**	***R*_Polar_**
**10** ^a)^	21.7	32.1	11.4	**10**	20.5 ± 4.6	39.5 ± 11.3	10.5 ± 2.0
**60**	20.0 ± 2.0	34.9 ± 2.5	12.0 ± 1.0	**60**	20.8 ± 6.2	37.9 ± 14.6	9.5 ± 2.0
**180**	20.9 ± 0.6	39.2 ± 0.9	11.3 ± 0.3	**180**	24.1 ± 2.5	46.1 ± 3.6	9.4 ± 0.7
**600**	21.4 ± 0.3	45.5 ± 0.4	10.9 ± 0.1	**600**	21.4 ± 0.3	45.5 ± 0.4	10.9 ± 0.1
**900**	21.3 ± 0.3	48.6 ± 0.4	11.0 ± 0.2	**900**	21.3 ± 1.0	49.4 ± 1.7	10.7 ± 0.5
**1200**	21.1 ± 0.4	48.5 ± 1.0	11.0 ± 0.2	**1200**	21.4 ± 0.9	51.5 ± 1.6	10.2 ± 0.5
**1500**	21.7 ± 0.4	51.9 ± 0.9	10.8 ± 0.2	**1500**	23.2 ± 0.5	52.9 ± 1.0	9.3 ± 0.4
**2100**	21.3 ± 0.3	54.1 ± 0.5	11.0 ± 0.2	**2100**	23.4 ± 1.1	57.5 ±1.5	9.7 ± 0.5
**2400**	22.1 ± 0.3	54.7 ± 0.5	10.5 ± 0.2	**2400**	22.1 ± 0.3	54.7 ± 0.5	10.5 ± 0.2
**2700**	21.1 ± 0.3	56.4 ± 0.6	11.2 ± 0.2	**2700**	22.5 ± 1.1	53.2 ± 1.7	9.5 ± 0.5
**3300**	20.8 ± 0.3	54.2 ± 0.5	11.0 ± 0.2	**4500**	23.2 ± 2.0	61.7 ± 3.3	9.9 ± 1.0

^a)^ Parameter values for the 10 s time point for the mixture of 150 mm 2C6PC + 0.25 mg mL^‐1^ PLA could not be determined reliably by optimization owing to the poor statistics, and the values presented are those determined by manual fitting to the measured data.

At the very early stages of the lipid degradation (*t* = 10 s), the system containing 150 mm 2C6PC exhibited micelles of similar structure to those seen in the absence of PLA2. Whereas, the micelles in the system containing 75 mm 2C6PC had already become longer (i.e., an increase in the length of the major equatorial radius) although significantly the thickness remained unchanged. At longer times of incubation, both the minor equatorial and polar radii were unchanged, while the major equatorial radius increased such that it almost doubles its original length at the longest incubation times. In both cases the half‐thickness of the lozenge remained fairly constant st ≈10.5 Å, the thickness of the 2C6PC molecule.

### 2.3 Molecular Dynamics Simulations

**Figure 3:**
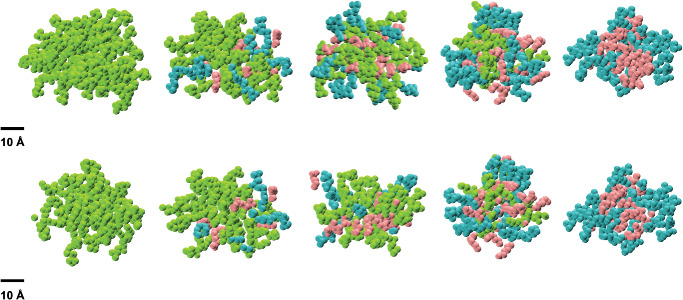
Snapshots of the micelles in the final frames of each trajectory. 2C6PC (Green), C6LYSO (Blue), C6FA (Pink) for simulations C6, C6‐25, C6‐50, C6‐75, and C6‐100 (top row, left to right). Also, the internal structures of the C6, C6‐25, C6‐50, C6‐75, and C6‐100 micelles (bottom row, left to right) obtained by cutting the micelles in the top row in half.

We have used MD simulations of the systems summarized in **Table** **3** to obtain a detailed description of the micellar structures formed in each system. The system labels (C6, C6‐25, C6‐50, C6‐75, and C6‐100) were assigned according to the percentage of 2C6PC that has been degraded. For example, in C6 there has been no degradation of 2C6PC whilst in C6‐25, 25% of the 2C6PC has been degraded into the corresponding C6LYSO and C6FA and so on. Visual analysis of the simulations shows that each system formed a single, stable micelle within the first 40 ns of the simulation. The formation of a single, stable micelle shows that the molecules self‐assemble in the simulations at a concentration above their CMC and within the range of proposed aggregation numbers, suggesting that the simulations accurately replicated the chemical nature of the lipids. **Figure** **3** shows snapshots of the equilibrated micelle found in each of the simulated systems.

**Table 3:** Composition of each system simulated. 2C6PC ‐ phosphocholine, C6LYSO ‐ lysophosphocholine, C6FA ‐ fatty acid

System	2C6PC	C6LYSO	C6FA	Total
C6	35 (0.27 M)	0	0	35
C6‐25	26 (0.2 M)	9 (0.07 M)	9 (0.07 M)	44
C6‐50	17 (0.13 M)	18 (0.14 M)	18 (0.14 M)	53
C6‐75	9 (0.07 M)	26 (0.2 M)	26 (0.2 M)	61
C6‐100	0	35 (0.27 M)	35 (0.27 M)	70
LYSO	0	35 (0.27 M)	0	35
FA	0	0	35 (0.27 M)	35

**Figure 4:**
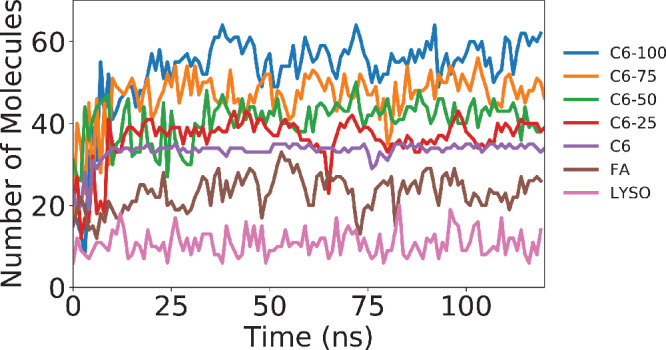
Micelle aggregation number over time for each simulated system.

By plotting the aggregation number of the largest micelle formed as a function of time for each system, the point at which stable micelles form can be determined (**Figure** **4**). The structure, size and composition of these micelles varied. The aggregation number of the stable micelle increases as the product concentration increases since a single 2C6PC molecule forms two product molecules.

### 2.4 Mixed Micelle Composition

One aim of this investigation was to understand how the presence of the products impacts the 2C6PC micelle. Therefore the composition of each micelle was closely scrutinized and compared. Some variation in the aggregation number of micelles over time is to be expected as, by their definition, micelles exist in a dynamic equilibrium with the monomers in solution. This can be best seen by comparing the standard deviations in the average aggregation number of the simulations between all systems. These standard deviations increase as the product concentrations increase confirming that higher product concentrations result in more dynamic, and potentially less stable, micelles.

It is important to note that the simulations of the pure C6 system resulted in the formation of a single micelle with an aggregation number of 34 ± 1, as suggested by earlier SANS studies.^[^ ^17^ ^]^ This suggests that the results of the MD simulations are indeed more in line with the triaxial model reported here than the prolate ellipsoidal model detailed previously.^[^ ^16^ ^]^

**Table** **4** shows the average number of each species within the micelle. It is clear that the vast majority of 2C6PC lipid molecules remained as part of the micelle in all simulations whilst the number of C6LYSO and C6FA molecules varied, with consistently less C6LYSO molecules found in the micelle than C6FA molecules. In all systems, we find that there are approximately double the number of C6LYSO molecules in the aqueous medium compared to C6FA which is consistent with previous work.^[^ ^19^ ^]^ In their work, Singh et al.^[^ ^19^ ^]^ examined the hydrolysis of DPPC vesicles by PLA2 and found that although equimolar quantities of the products were produced, twice as much LYSO product was found in solution compared to the FA. This observation is explained by considering the increased hydrophobicity of a FA molecule relative to a LYSO molecule. The nature of the hydrophobic effect means that a FA molecule is more likely to aggregate in an attempt to shield its hydrophobic carbon tail from water as it lacks the hydrophilic phosphocholine head group found on a LYSO molecule. This is consistent with the findings from our SANS experiments that revealed an increase in the major equatorial radius of the micelles during degradation. This result is a consequence of the C6FA degradation product remaining associated with the aggregates formed by 2C6PC while the more soluble C6LYSO, formed during hydrolysis by PLA2, has more tendency to be located in the protein matched water (PMW) as it is too hydrophilic to remain solely in the micelles.

**Table 4:** Average number and % of each type of molecule within the system that are found in the micelle over the last 120 ns

**Lipid**	2C6PC	C6LYSO	C6FA
**C6**	34 ± 1.0 (97%)	–	–
**C6‐25**	25 ± 1.4 (96%)	6 ± 1.5 (67%)	7 ± 1.3 (78%)
**C6‐50**	16 ± 0.8 (94%)	11 ± 2.2 (61%)	15 ± 1.6 (83%)
**C6‐75**	9 ± 0.5 (100%)	21 ± 2.4 (78%)	23 ± 1.9 (85%)
**C6‐100**	–	30 ± 2.6 (86%)	33 ± 1.5 (94%)
**FA**	–	–	31 ± 1.0 (74%)
**LYSO**	–	0 (0%)	–

The C6‐100 simulation resulted in the formation of mixed micelles. Whilst the CMC of the C6LYSO/C6FA mixture is unknown, it is expected^[^ ^19^ ^]^ that the C6LYSO/C6FA solution will have a CMC that is higher than the respective solution of 2C6PC molecules. Therefore, the highly concentrated simulation, which is ≈20 times the 2C6PC CMC, is likely to meet this threshold.

### 2.5 Mixed Micelle Properties

The properties of each micelle were examined to obtain information concerning the size (radius of gyration) and the shape (moments of inertia) of the micelles. From these quantities, we then estimated the micellar radius to determine the size of the micelles and the eccentricity to determine the shape of the micelles. We have also documented the length of the largest dimension in each micelle by calculating the largest distance between two atoms in the micelle. This allows us to compare the relative changes in the size of the micelles in the simulations to changes in the micelle dimensions obtained when modeling the SANS data.

**Figure 5:**
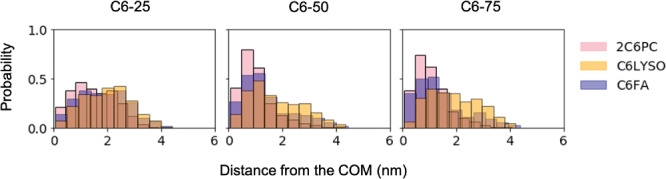
Distribution of different lipid components from the micelle center of mass (COM). Determined by calculating the distance of the furthest atom in each 2C6PC, C6LYSO, and C6FA molecule from the micelle COM and then normalizing to 1 for each molecular species.

The eccentricity values documented in **Table** **5** show that the micelles are not spherical as spherical micelles have an eccentricity of 0. By comparing the moments of inertia, the micelle shape can be classified as either prolate (one major and two minor axes) or oblate (one minor and two major axes) using the criteria defined within the Supporting Information. Whilst the micelles appear closest to a prolate shape based on the eccentricities, I1 ≠ I2 ≠ I3 which suggests that the simulated micelles are in fact triaxial. The triaxial shape of the micelle, whilst conflicting with previous experimental^[^ ^16^ ^]^ and simulation^[^ ^20^ ^]^ studies, does agree with the SANS modeling detailed within this paper. The changes in the I1/I2 and I2/I3 ratios with degradation (as seen in Table 5) warranted further investigation as these indicate changes to the internal structure of the micelle. **Figure** **5** shows the distribution of 2C6PC, C6LYSO, and C6FA throughout the micelles. These distributions illustrate that the 2C6PC and C6FA molecules can be found clustered in the center of the micelle whilst the more hydrophilic C6LYSO molecules, which have a broad distribution throughout the micelle, are present at the surface of the micelle.

**Table 5:** Analysis of micelle properties over last 100 ns of simulation

System	R_*g*_(Å)	Length (Å)	I1/I2	I2/I3	Eccentricity
C6	17.4 ± 0.8	49.5 ± 1.6	0.59	0.91	0.34
C6‐25	18.3 ± 1	49.0 ± 2.2	0.63	0.90	0.31
C6‐50	18 ± 1	48.6 ± 2.0	0.71	0.88	0.25
C6‐75	16.4 ± 1	48.3 ± 2.5	0.73	0.88	0.24
C6‐100	17 ± 1	48.8 ± 2.2	0.73	0.88	0.23
FA	9.9 ± 2	0	0.64	0.87	0.31

The micellar radii of gyration, as shown in Table 5, show no obvious trend other than increasing standard deviation as the percentage of the product molecules increases. This is likely linked to the increasingly dynamic nature of the micelles as the amount of product molecules present within them increases, as shown in Figure 4. The values are also similar to the *R*_g_ obtained from the SANS data detailed previously, suggesting good agreement between the SANS and the simulation data.

The maximum length of the micelles within the simulations is larger than the major equatorial radius of the micelles obtained from the SANS data (Tables 1 and 2). However, the length values calculated from the MD trajectories refer to the largest distance between any two atoms within the simulation. This is not an equivalent measurement to the radius obtained from the SANS data, which would naturally be smaller than the largest distance between two atoms within the micelle. Instead we use the maximum length value to monitor the same trends in micelle shape within the simulation that we use the radius to follow within the SANS.

Throughout the simulated degradation process (C6 → C6‐25 → C6‐50 → C6‐75 → C6‐100) there is very little change in the maximum length of the micelles as the 2C6PC is degraded. This is because within the simulation there is a finite number of C6FA molecules and they are all derived from the 2C6PC molecules within the original micelle. The SANS results (Figure 2 and Table 2) show as the micelle degrades, the resulting 2C6PC/C6FA/C6LYSO micelle increases in size along the major equatorial radius and we note that this is due to the incorporation of the C6FA degradation products from other micelles within the system. In order to test this within the simulation we performed two further simulations, detailed in Table S1, Supporting Information, where we increased the number of 2C6PC, C6LYSO and C6FA molecules available for the C6‐25 and C6‐75 systems. In the simulations where the number of the C6FA molecules and C6LYSO molecules was increased, the maximum length also increased, as did the number of molecules within the primary cluster. This supports the assertion of the SANS experiments that the micelles increase in size during degradation due to incorporation of additional C6FA within the resulting micelles.

### 2.6 Phosphocholine Hydration

The hydration of the PC lipid molecules was characterized by calculating the coordination of water molecules around selected atoms within the lipid head group for both lipid molecules existing as isolated monomers or in the micelle (Table 4). As there is no record of previously documented hydration numbers for the C6PC lipid molecule, we have used the hydration numbers obtained for monomeric 1,2‐dipropionyl‐sn‐glycero‐3‐phosphocholine (C3PC) lipids^[^ ^21^ ^]^ using simulations and neutron diffraction experiments for comparison. In that study the nitrogen (N) atom in the phosphocholine head group of the C3PC monomer was hydrated by 21 water molecules which is identical to the 21 water molecules found to hydrate the N atom of the C6PC monomer in this study. Although there is some evidence^[^ ^21^ ^]^ of phophocholine hydration decreasing as chain length increases, the difference in size between the C3PC and C6PC is likely too small to reflect this.

The g
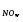
(r) (distribution of the water oxygen O_w_ around the phosphocholine nitrogen atom, N) and g
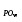
(r) (distribution of the water oxygen O_w_ around the phosphocholine phosphate atom, P) are nearly identical for all of the PC‐containing systems studied in this manuscript. This suggests that the presence of the products is not significantly altering the interactions of water with the phosphocholine head group of the parent lipid molecules.

Analysis of the number of water contacts across the entire simulation revealed a slight difference in the hydration of the head group if it exists as a monomer or part of the micelle. The difference in hydration corresponds to one less water molecule hydrating the head group when the lipid is part of the micelle as opposed to when it exists as a monomer. This small difference between monomers and micelles is not surprising as even when the phosphocholine lipid molecule exists as part of the micelle, its hydrophilic nature will attempt to maximize interactions with water. This is in line with a study^[^ ^22^ ^]^ which showed an increase in hydration of C6LYSO monomers as compared to micelles.

The slight change in head group hydration is in contrast to the hydration of the hydrophobic terminal carbons which show a drastic change between lipid molecules in a micelle and monomers. An example of this can be seen in **Table** **6** which shows the number of water molecules associated with N, P, and the terminal carbons. An increase in the hydration of the terminal carbons for monomeric lipids is expected as they are not shielded from interactions with water molecules by surrounding lipid molecules. The significant difference shown between the hydration of C26 and C36 in the micelles and monomers illustrates the amount of shielding offered to the hydrophobic carbon chains in micelles.

**Table 6:** Number of water contacts made with each atom in all systems, both as monomers and when they exist as part of the micelle

Molecule	Atom	C6	C6‐25	C6‐50	C6‐75	C6‐100	Monomer
2C6PC	N	20	20	20	20	–	21
	P	8	8	8	8	–	8
	O22	1	1	1	1	–	2
	O32	1	1	1	1	–	2
	C26	4	4	5	4	–	17
	C36	4	4	4	4	–	19
C6LYSO	N	–	20	20	20	20	21
	P	–	7	7	7	7	8
	O	–	1	1	1	1	2
	C	–	6	6	6	6	17
C6FA	O1	–	2	2	2	2	4
	O2	–	4	4	4	4	7
	C	–	3	3	3	4	15

The 2C6PC monomer has an additional hydrating water molecule in the first hydration shell for both of the double bonded oxygens in the ester groups on both tails compared to the 2C6PC in the micelle. This suggests that the ester group is at least partially shielded when in the micelle. Empirical potential structure refinement (EPSR) modeling of neutron diffraction data for the C3PC lipids in solution^[^ ^21^ ^]^ gave a hydration number of ≈2 at a distance of 2.5 Å whilst we recorded a hydration number of ≈2 at 3.2 Å which corresponds to the first minimum in the O−O_w_ RDF as seen in Figure S4, Supporting Information.

### 2.7 Lysophosphocholine Hydration

When part of a micelle, the hydration of the terminal carbon in the C6LYSO molecules is larger than for 2C6PC terminal carbons. This trend is the result of a reduction in hydrophobic repulsion and steric hindrance around the terminal carbons due to the absence of the additional hydrocarbon tail. Consequently, C6LYSO molecules are less inclined to embed deeply into the micelle due to their reduced hydrophobic nature. This is demonstrated in Figure 5 which shows the distance of each lipid type from the micelle center of mass (COM).

The hydration of the ester C=O oxygen is identical to the hydration seen for the same group in the 2C6PC lipid molecules, with each oxygen being hydrated by a single water molecule in the micelle but hydrated by two water molecules when they are monomers. This suggests that the micelle continues to partially shield the ester group of the C6LYSO molecules from water molecules. Also, it implies that the loss of an additional carbon chain does not result in significant changes to the arrangement of water around the ester region. Whereas, the phosphate group of the C6LYSO molecules in a micelle are hydrated by one less water molecule than the phosphate groups of C6PC molecules that are a part of a micelle. However, this difference is within the standard deviation calculated and therefore may not be significant.

### 2.8 Fatty Acid Hydration

As with 2C6PC and C6LYSO, the C6FA experiences very little change in the hydration of either the terminal carbon or the oxygens that compose the carboxylic acid head group throughout the degradation process. There is a significant increase in the hydration of the terminal carbon between the monomeric and micellular species which is also something seen with the 2C6PC and C6LYSO molecules and it suggests that the hydrocarbon tail of the FA exists within the core of the micelle where it is shielded from water. It is also interesting to note the significant difference in the hydration of monomeric oxygens of the C6FA compared to those in the micelle. The large increase in surrounding water molecules suggests that the C6FA head group is somewhat shielded from the aqueous solvent when it is part of a micelle. The consistency of the O1 and O2 hydration throughout the degradation process suggests that 2C6PC is not solely responsible for the shielding of the C6FA head group as the hydration remains unchanged in the C6‐100 system, where there is no 2C6PC.

Considering Figure 5 which suggests that the C6FA is found predominantly in the center of the micelle, whilst C6LYSO is found closer to the surface, we suggest that the hydrophobic tails of the C6FA and 2C6PC form the core of the micelle in 2C6PC/C6LYSO/C6FA mixed micelles and that the C6FA hydrophobic tails form the core within the C6LYSO/C6FA mixed micelle. Interdigitation of the C6LYSO hydrophobic tail partially into this hydrophobic core would account for the slightly increased hydration of the C6LYSO terminal carbons when compared to the 2C6PC and C6FA terminal carbons which are found in the center of the micelle and are therefore more shielded from the aqueous environment. It would also explain the shielding the C6FA head group as the much larger head group of the C6LYSO would then sterically hinder interactions between the C6FA oxygens and water.

### 2.9 Pure Fatty Acid and Lyso‐PC Micelles

**Figure 6:**
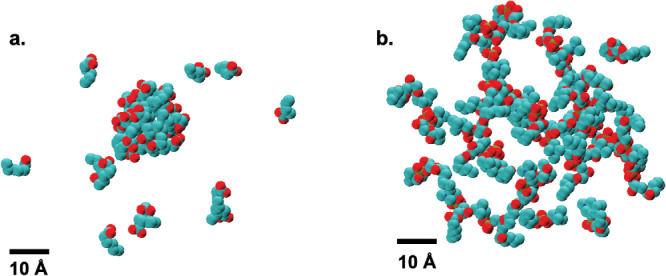
Snapshots of the final frames for a) the FA system, showing both the micelle and monomers and b) the LYSO system. The carbons are colored in cyan, the oxygens red, the phosphorous in yellow and the nitrogens in dark blue.

The simulation of the FA system containing only C6FA molecules resulted in the formation of a single stable micelle with an average aggregation number of 31 ± 1. The micelle exists in a dynamic equilibrium with the C6FA monomers in solution however a single micelle remains present for the duration of the simulation. Whereas upon visual inspection of the LYSO system, we observe that the molecules do not form a micelle, but instead they form a network of loosely associated molecules, as seen in **Figure** **6**. Previously reported trends in the CMC of lyso‐PC molecules^[^ ^23^ ^]^ suggests the CMC of the C6LYSO molecules would be significantly higher than the concentration at which they are found in the simulation. The fact that the C6LYSO molecules are unable to form a stable micelle would be consistent with these findings.

## 3 Conclusion

By using MD simulations in conjunction with SANS experiments we determined that a triaxial model was a better representation of 2C6PC micelles than a prolate ellipsodal or spherocylindrical model. This is a key finding as a range of aggregates of very different dimensions and aggregation numbers have been previously proposed when analysing the SANS data. Additionally, this finding demonstrates that not only are MD simulations useful in gaining molecular scale detail of these systems, but are also very useful in fitting the experimentally derived data in these complex systems.

Categorizing each individual molecule as either a monomer or a component of a micelle revealed the relative likelihood of the two products being found in the micelle is directly correlated with their hydrophobicity. We have also, for the first time, detailed the hydration of the 2C6PC and C6LYSO lipids and illustrated the degree to which micellization alters this hydration. This has allowed us to better understand the driving forces of micellization for these species as we have shown that the presence of two hydrocarbon chains, as in 2C6PC, is required to stabilize the micelles. Upon degradation of 2C6PC, the tails of the more hydrophobic C6FA form the core of the micelle and C6LYSO tails interdigitate partway into that hydrophobic core. Therefore, as the relative amount of C6LYSO to 2C6PC increases the micelles become more dynamic, as shown by Figure 4, but are still intact.

This work has addressed key questions regarding the behavior of the 2C6PC, C6LYSO and C6FA species before, during and after degradation. This was achieved by showing that upon degradation, dynamic and stable mixed‐micelles composed of 2C6PC, C6LYSO, and C6FA formed. When total degradation of 2C6PC species occurred, a C6LYSO/C6FA mixed micelle formed. Closer study of the stable C6LYSO/C6FA micelle revealed no significant changes to the hydration of the C6LYSO or C6FA carbon tails when compared with other micelles suggesting the retention of a hydrophobic core.

## 4 Experimental Section

### 4.1 Materials

1,2‐dihexanoyl‐sn‐glycero‐3‐phosphocholine, 2C6PC, (>99% purity) and 1‐hexanoyl‐2‐hydroxy‐sn‐glycero‐3‐phosphocholine, C6LYSO, (> –99% purity, may contain up to 10% of the 2‐LYSO isomer) were purchased from Avanti Polar Lipids (Alabama, USA). D_2_O (99.9 atom % D), hexanoic acid, C6FA, and phospholipase A2 (PLA2) from bovine pancrease (P6534) were obtained from Sigma‐Aldrich. All other materials were obtained from Sigma‐Aldrich and were of the highest grade available. Molecular structures of the 2C6PC, C6LYSO and C6FA can be found in Figure S1, Supporting Information.

### 4.2 Small‐Angle Neutron Scattering

The SANS experiments were performed using the SANS2D time‐of‐flight beamline at the ISIS pulsed neutron source (STFC Rutherford‐Appleton Laboratory, UK). SANS2D utilizes neutrons with wavelengths of 2–14 Å that are measured on a 96.5 × 96.5 cm^2^, 2D detector at a distance of 4 m from the sample, giving a scattering vector, *Q* = (4*πλ*) sin(θ/2) in the range of 0.0045 ≤ *Q* ≤ 0.4 Å^−1^. Wavelength‐dependent corrections are performed to correct for the incident spectrum, detector efficiencies, and measured sample transmissions to create a composite SANS pattern. The resulting SANS data was then placed on an absolute scale (cm^−1^) using the scattering from a standard sample of a solid blend of hydrogenous and perdeuterated polystyrene in accordance with established procedures. All SANS measurements were performed at 298 ± 0.1 K. The SANS2D experiments were routinely performed in event mode, a technique which allows for the retrospective analysis (i.e., time slicing) of the SANS data.

In SANS experiments in which the PLA2 enzyme was present, it was necessary to ensure that the scattering length density of the H_2_O:D_2_O solvent (ρ_s_) matched the scattering length density of the bovine pancreatic phospholipase A2 enzyme (ρ_PLA2_) (assumed here to be 2.68 × 10^−6^ Å ^−2^) in order to determine the scattering due only to 2C6PC and any degradation products. Under these ‘contrast match’ conditions (where ρ_PLA2_‐ρ_s_ = 0), the PLA2 enzyme did not contribute to the small angle scattering measured. The contrast match point of the PLA2 enzyme was predicted from a knowledge of the amino acid composition of the protein and was determined to be 46.6 vol% D_2_O (Biomolecular Scattering Length Density Calculator). This is referred to as PMW (note that the scattering length density of D_2_O = 6.35 x 10^−6^ Å ^−2^ and that of H_2_O = − 0.56 × 10^−6^ Å ^−2^.).

#### 4.2.1 Static Small Angle Neutron Scattering Measurements

For the static SANS measurements, sample dispersions, and their corresponding solvents, were placed in 2 mm path length disk‐shaped quartz cuvettes (Hellma, U.K., Ltd., Essex) and measured using a 12 mm diameter neutron beam. To calculate the excess scattering arising solely from the sample, the scattering from the appropriate solvent was subsequently subtracted from the sample data sets.

Subsequently, the SANS profiles of dispersions of 2C6PC were recorded at a range of concentrations, namely 30, 75, and 150 mm (i.e., approximately two, five, and ten times the CMC),^[^ ^4^, ^18^ ^]^ dispersed in either D_2_O or D_2_O containing Tris buffer (10 mm Tris) at a pD equivalent to a pH of either 7.4 or 8.0 (adjusted with HCl) with 150 mm NaCl and 5 mm CaCl_2_.

#### 4.2.2 Time‐Resolved Small Angle Neutron Scattering Measurements

Time‐resolved SANS experiments were performed in PMW containing TRIS buffer, 150 mm NaCl and 5 mm CaCl_2_ at pH 7.4. Prior to performing the stopped flow SANS measurements, the ability of the PMW to match the protein was confirmed by determining the scattering using the highest concentration of protein used which was found to be indistinguishable from the PMW alone.

Once the ability of the D_2_O:H_2_O solvent to contrast match the PLA2 enzyme was determined, the same composition aqueous solvent containing Tris buffer (10 mm Tris) at a pD equivalent to a pH of 7.4 (adjusted with HCl) with 150 mm NaCl and 5 mm CaCl_2_ was used to prepare stock solutions of 2C6PC micelles and PLA2 at twice the final strength required so that once mixed in equal volumes in the Biologic Co SFM‐3 stopped‐flow apparatus (Claix, France), fitted with Bio‐Kine software (Claix, France), the concentration obtained was as required for the study. The Biologic Co SFM‐3 stopped‐flow apparatus was fitted with a neutron‐scattering observation head containing a rectangular cell of 1 mm path length which allowed an 8 mm diameter neutron beam to be utilized. All time‐resolved SANS experiments were performed in event mode with a total counting time of 4500 s. After the appropriate solvent background was subtracted from all the sample sets, the SANS data were retrospectively analyzed (i.e., time sliced) depending upon the kinetics of the degradation process.

#### 4.2.3 Analysis of SANS Data

All fitting procedures of the SANS data were performed using the SASVIEW package^[^ ^24^ ^]^ using a range of models (*P*(*Q*)) including spheroidal (oblate, prolate, sphere), capped cylindrical and triaxial micelles. Due to the high concentrations of 2C6PC studied, it was necessary to account for any interparticulate interactions (*S*(*Q*)), which was achieved using a hard sphere model. The modeling assumed a flat background correction to allow for any mismatch in the incoherent and inelastic scattering between the samples and solvent. The fitted background levels were always checked to ensure that they were of a physically reasonable magnitude.

When analyzing the SANS data, the (extended) length of the hydrocarbon tails (R_*tail*_) of the 2C6PC molecules that comprise the hydrophobic “core” of the micelle was estimated using Tanford's formula (R_*tail*_ = (1.5 + 1.265*n*) Å where *n* = the number of carbons in the core).^[^ ^25^ ^]^ In the present study the “core” was assumed to be composed of the last five carbons of the hydrocarbon tails with the ester groups being considered to be part of the hydrophilic head group contained within the micelle “shell”. The length of the hydrophobic tail was thus calculated to be 7.8 Å. However, it should be noted that the hydrocarbon chains in a micelle are generally in a “fluid‐like” rather than “extended, solid‐like” crystalline nature. Consequently, the effective length of the five carbon tail in the 2C6PC micelles is likely to be less than the fully extended length of 7.8 Å estimated using the Tanford calculation. Generally this is assumed to be ≈75–80% of the extended length, that is, a value in the range of 5.9–6.2 Å. In all cases, the thickness of the shell region was fixed at 5 Å. The volume of the hydrophobic chains of the lipid (*V*_tails_) was calculated using Tanford's formula (*V*_tails_ = (54.8 + 2 × 26.9*n*) Å^3^ where *n* = 5) to be 323.8 Å^3^.

### 4.3 Molecular Dynamics of Phosphocholine Self‐Assembly

Atomistic MD simulations were used to investigate the formation and degradation of micelles containing 2C6PC in an aqueous solution. All simulations were carried out using the Gromacs v5.1.2 MD simulation package.^[^ ^26^, ^27^, ^28^ ^]^ The 2C6PC molecules used in the simulations were created and minimized using Avogadro.^[^ ^29^ ^]^ The molecules underwent energy minimization to prevent local structural distortion.

An initial simulation system was set up which consisted of 35 2C6PC molecules and 6475 water molecules (1:185). This corresponds to a concentration of 0.3 M which was ≈20 times the reported CMC of the lipid (14 mm),^[^ ^30^ ^]^ it has previously been shown that the physical properties of the micelles formed by 2C6PC molecules are not sensitive to concentration. This number of lipids also allowed us to have approximately twice the minimum aggregation number (19 molecules) documented in the literature.^[^ ^12^, ^16^ ^]^ The molecules were randomly positioned within the box, the system was minimized using a steepest descent algorithm and then thermalized at a temperature of 300 K for 20 ps using the Nose–Hoover thermostat. After thermalizing, the systems were equilibrated to a constant pressure using an NPT ensemble which maintained a constant number of particles (dependent on the system), a constant temperature (300 K) and a constant pressure (1 atm). The system was equilibrated for 2 ns and then the production simulation was run for 140 ns.

### 4.4 Molecular Dynamics of Phosphocholine Products

Since the MD simulations run for these systems used a classical force field, the simulations were unable to model the bond formation and bond breakage which occur during lipolysis. Therefore, the 2C6PC lipid cannot be degraded and the products cannot be formed whilst the simulation is running. In order to investigate the impact of PLA2 digestion on the micelles, six further simulations were conducted where both parent and product (C6LYSO and C6FA) molecules began the simulation in a random arrangement within the box. This was in contrast to the real system where an existing micelle is hydrolyzed by PLA2. Nevertheless, this setup allows the interactions between the parent and product molecules to be studied as well as the structure and the dynamics of the aggregates they form. This was done to mimic the destruction of the micelle and the formation of the products by PLA2 over time. In addition to this, systems that contained only the C6LYSO product and only the C6FA product were also simulated. The systems, detailed in Table 3, contained different ratios of parent to product molecules so the behavior of the system at different stages of degradation was able to be investigated.

The C6LYSO and the C6FA products were built using Avogadro and parameterized using the CHARMM General Force Field (CGenFF)^[^ ^31^, ^32^ ^]^ program which generated atom types and parameters for each molecule.^[^ ^33^ ^]^ All of the reported ‘penalties’ were lower than ten which indicated that the assignments were reliable and could be used in the MD simulations.

The systems in Table 3 were minimized, thermalized and equilibrated following the same protocols as the pure C6 system. After these initial simulation stages, the production simulations were run for 120 – 140 ns.

## Conflict of Interest

The authors declare no conflict of interest.

## Supporting information

Supporting Information
