# Supplementary material for: The Impact of Lipid Digestion on the Dynamic and Structural Properties of Micelles
Source: Small. 2021 Jan 20;17(6):2004761. doi: 10.1002/smll.202004761 (PMC11475325; doi:10.1002/smll.202004761)
Supplement: Supplementary file 1 — Supporting Information [file SMLL-17-2004761-s001.pdf]

© 2021 Wiley-VCH GmbH

small NANO MICRO

Supporting Information

for *Small*, DOI: 10.1002/smll.202004761

The Impact of Lipid Digestion on the Dynamic and Structural Properties of Micelles

Demi L. Pink, Fabrizia Foglia, David J. Barlow, M. Jayne Lawrence, and Christian D. Lorenz**

Supporting Information for "The impact of lipid digestion on the dynamic and structural properties of micelles"

Demi L. Pink,[†] Fabrizia Foglia,[‡] David J. Barlow,[¶] M. Jayne Lawrence,[*,§] and Christian D. Lorenz[*,†]

†*Department of Physics, King's College London, London, WC2R 2LS, UK*

‡*Department of Chemistry, University College London, 20 Gordon Street, London WC1H 0AJ, UK*

¶*Faculty of Life Sciences and Medicine, King's College London, Franklin-Wilkins Building, Stamford Street, London SE1 9NH, UK*

§*Division of Pharmacy and Optometry, School of Health Sciences, Faculty of Biology, Medicine and Health, University of Manchester, Stopford Building, Oxford Road, Manchester, UK*

E-mail: jayne.lawrence@manchester.ac.uk; chris.lorenz@kcl.ac.uk

Methods

Molecular structures

Figure S1 shows the molecular structures of the three species (a phosphocholine, a lysophosphocholine and a carboxyilic acid) investigated within this work. PLA2 refers to the phospholipase A2 enzyme involved in the hydrolysis of the sn-2 acyl bond however other phos-

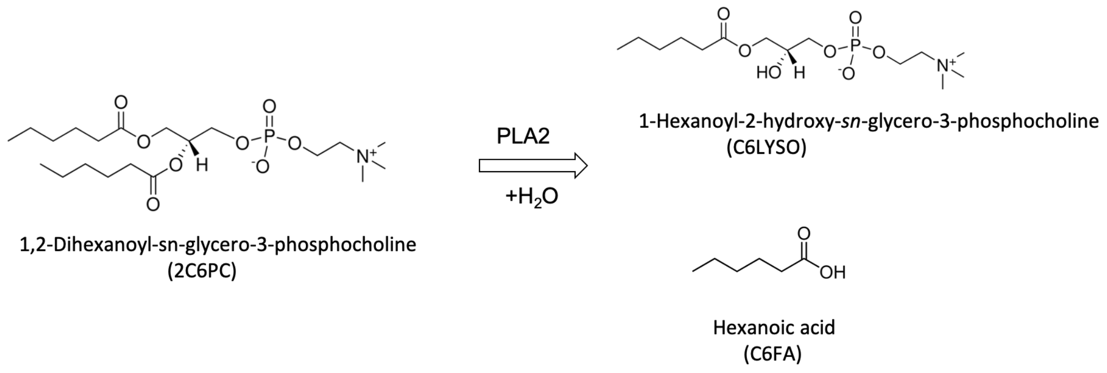

Figure S1: The structure of the parent 2C6PC molecule alongside the structures of the C6LYSO and C6FA productmolecules following degradation by the PLA2 enzyme in aqueous solution.

pholipase enzymes do exist. These phosholipase include PLA1 which cleaves at the sn-1 position, PLB1 which can cleave at both the sn-1 and sn-2 positions and PLC which cleaves the phoshocholine at the phosphate group.

Analysis of simulations

Analysis of the simulated systems was conducted using a combination of GROMACS tools and specific, purpose built tools developed in-house. The analysis was performed over the trajectory following the equilibration of the micelles, as determined by monitoring micelle size over time.

Radial distribution functions (RDFs) define the probability of finding a particle at a distance, r, from a reference particle. The average number of the particles at distance r from a reference particle, also known as the coordination number ($n_c(r)$), was determined by integrating under the RDF curve from 0 to r. Generally, the value of $n_c(r)$ where r is equal to the first minimum in the RDF is used to determine the average number of particles within the first neighbor shell of a reference particle.

One of the aims of this investigation was to determine the differences between lipids that existed as a part of the micelle compared to isolated lipids in solution so it was important to determine the time points where each individual lipid molecule was within a micelle.

From visual inspection of the trajectories using VMD, it was clear that a single large micelle was formed in each trajectory along with a number of monomers isolated in solution with no secondary aggregates being formed. As a result, a tool was developed which defined a molecule as being part of the micelle if the terminal carbons of that lipid were within the first neighbor distance (4 Å) of another lipid's terminal carbons. This value for identifying aggregated lipids, which was determined from the RDF shown in Fig. S3, was validated by visual examination of the trajectory which showed that smaller values excluded lipid molecules that were seen to be part of the micelle and larger values included lipid monomers as part of the micelle.

To assess the shape of the micelles, the radius of gyration, moments of inertia and eccentricity were determined for the last 100 ns of each simulation. These values only provide useful information when calculated with regards to a specific, single micelle or object and cannot describe the entire dynamic system.

The moments of inertia measure the distribution of the mass of an object relative to a given axis. By understanding how mass is distributed throughout the micelle, we are able to determine the shape of the micelle. Here the moments of inertia are defined as:

$$\mathrm{I1} < \mathrm{I2} < \mathrm{I3}$$

By examining the ratios I1/I2 and I2/I3 the shape can be described so that:

$$\mathrm{I1} = \mathrm{I2} = \mathrm{I3} \rightarrow \mathrm{I1/I2} = 1, \mathrm{I2/I3} = 1 \rightarrow \text{Spherical}$$
$$\mathrm{I1} = \mathrm{I2} < \mathrm{I3} \rightarrow \mathrm{I1/I2} = 1, \mathrm{I2/I3} = 0 \rightarrow \text{Oblate}$$
$$\mathrm{I1} < \mathrm{I2} = \mathrm{I3} \rightarrow \mathrm{I1/I2} = 0, \mathrm{I2/I3} = 1 \rightarrow \text{Prolate}$$

Determining the moments of inertia allows a micelle's eccentricity to be calculated using the equation:

$$\text{Eccentricity} = 1\text{-}(\mathrm{I}_{min}/\mathrm{I}_{ave})$$

where I_{min} is the average of I1 and I_{ave} is the average across all moments of inertia. An eccentricity value of 0 means that a micelle is completely spherical and so eccentricity can be used to measure the degree to which a micelle deviates from a perfect sphere.

Further 2C6PC simulations

In addition to simulating the degradation of a single micelle, two further simulations were performed. These simulations are the F-C6-25 and the F-C6-75 which were simulated in order to understand the impact of additional 2C6PC, C6FA and C6LYSO on the size and dynamics of the degrading micelles.

Simulations of the F-C6-25 and F-C6-75 systems were performed by extracting the final frame of the original C6-25 and C6-75 simulations. Additional 2C6PC, C6FA and C6LYSO molecule were added in the appropriate ratios, corresponding to a starting micelle containing 53 2C6PC molecules as this is almost 50x the original number of 2C6PC molecules. Water was also added to ensure that the concentration remained $\approx$ 20x the CMC for both systems. The final number of 2C6PC, C6FA and C6LYSO in each system is detailed in Table S1. Both systems then underwent equilibration following the same protocol as is documented in the main paper, this ensured a temperature of 309K and a pressure of 1 atm. The systems were then simulated for a 40 ns.

Results

Further 2C6PC simulations

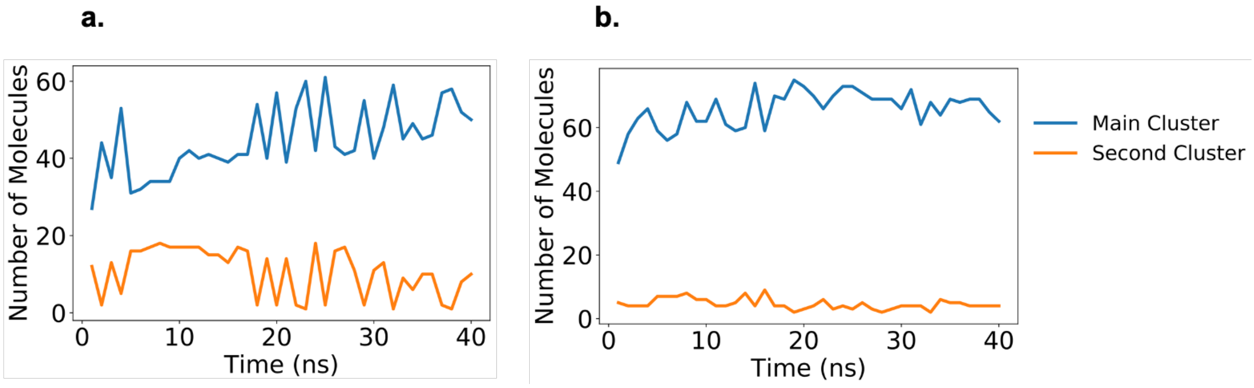

Figure S2: The aggregation numbers of the primary and secondary clusters overtime within a. the F-C6-25 system and b. the F-C6-75 system.

The aggregation of the F-C6-25 and F-C6-75 equilibrated within the fist 20ns of the simulation, as can be seem by following the aggregation numbers over time in Fig. S2. Both systems resulted in the the formation of two micelles, a large primary micelle and a much smaller secondary micelle as well as some lipids remaining in solution.

Table S1: Number of 2C6PC, C6LYSO and C6FA present with the entire system and the number of 2C6PC, C6LYSO and C6FA molecules contained within the primary cluster of both systems.

		2C6PC	C6LYSO	C6FA	Total
Whole system	F-C6-25	39	14	14	67
	F-C6-75	14	39	39	92
Primary cluster	F-C6-25	28	8	10	46
	F-C6-75	12	24	30	66

The secondary micelle within the F-C6-25 was very unstable and often attempted to coagulate with the primary cluster, forming a single large micelle. This can be seen in Fig. TK which shows the temporary formation of much larger cluster containing ≈ 60 molecules which corresponds with a loss of the secondary micelle. This is likely because there were too many 2C6PC molecules within the simulation to form a single, stable micelle but two few 2C6PC molecules to form two stable clusters.

This is supported by the formation of a large primary micelle in the F-C6-75 system along with a much smaller secondary cluster containing 5 lipids as this system only contained 14 2C6PC lipids. The primary cluster within this system consisted mainly of C6FA and C6LYSO, unlike the F-C6-25 primary cluster which contained mostly 2C6PC. The secondary cluster within the F-C6-75 system is so small as the C6LYSO is not hydrophobic and so is stable as a monomer in solution.

The average aggregation numbers of the primary clusters within the F-C6-25 and F-C6-75 systems are much larger than in the corresponding C6-25 and C6-75 systems with F-C6-25 containing 18 % more molecules and the F-C6-75 increasing by 25 %. Further details of the composition of each primary cluster can be found within Table S1. In addition, the maximum length of the F-C6-25 and F-C6-75 systems increases however the R_g values and

Table S2: Structural parameters of the primary clusters within the F-C6-25 and F-C6-75 simulations.

	R_g (Å)	Max. length (Å)	I1/I2	I2/I3
F-C6-25	17.4 ± 1.4	53.7 ± 3.4	0.61	0.86
F-C6-75	18.5 ± 2.3	55.0 ± 2.5	0.75	0.87

moments of inertia remain comparable to those in the original C6-25 and C6-75 simulations, these values are shown in Table S2.

Radial distribution functions

2C6PC RDF

In this study the RDFs of the 2C6PC carbon tails, (C_{26}-C_{26} and C_{36}-C_{36}), were calculated and plotted for all lipids in order to determine the distance parameter needed to classify lipids as either monomers or part of the micelle. Based on the RDF shown in Fig S3 and on visual analysis of the simulations, the distance of the first peak (≈ 4 Å) was used to estimate the minimum distance needed for a lipid to be considered part of the micelle. This meant that any lipid not within 4 Å of another lipid was considered a monomer.

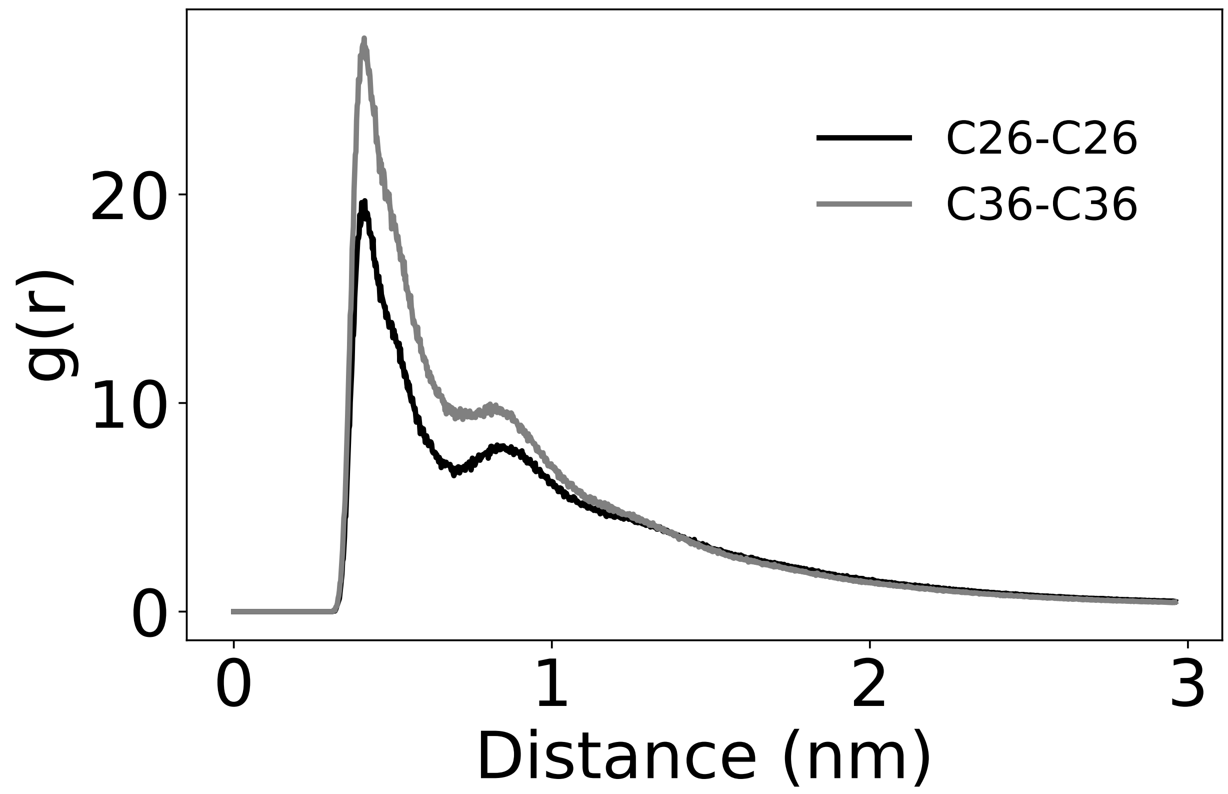

Figure S3: The RDF of the two terminal carbons with respect to all other terminal carbons (C_{26}-C_{26} and C_{36}-C_{36})

Water hydration

Table S3: The distance of the first minimum in the RDFs for atoms that were part of the micelle was used to determine the number of water contacts for atoms in both micelles and monomer system.

**System**		**Hydration shell (nm)**
2C6PC	N-Ow	0.574
	P-Ow	0.456
	C26-Ow	0.54
	C36-Ow	0.54
	O22-Ow	0.32
	O32-Ow	0.32
C6LYSO	N-Ow	0.574
	P-Ow	0.456
	C-Ow	0.546
	O-Ow	0.32
C6FA	O1-Ow	0.352
	O2-Ow	0.426
	C-Ow	0.518

The RDFs of the water oxygens (O_w) with respect to several selected atoms were recorded for lipids that were part of the micelle and for lipids that were monomers. Figure S4 show the RDFs for N-O_w, P-O_w, C_{26}-O_w, C_{36}-O_w, O_{22}-O_w and O_{32}-O_w when part of the micelle in the C6 system. This was done to determine the water hydration of the atoms in order to compare hydration between the different systems and between micelle/monomer systems. The hydration number was calculated using the coordination number obtain at the first minimum in each RDF plot which is detailed in Table S3. The distance of the first hydration shell determined for atoms when then formed part of the micelle was also the distance used to calculated the number of water contacts for the atomic constituents of the monomers.

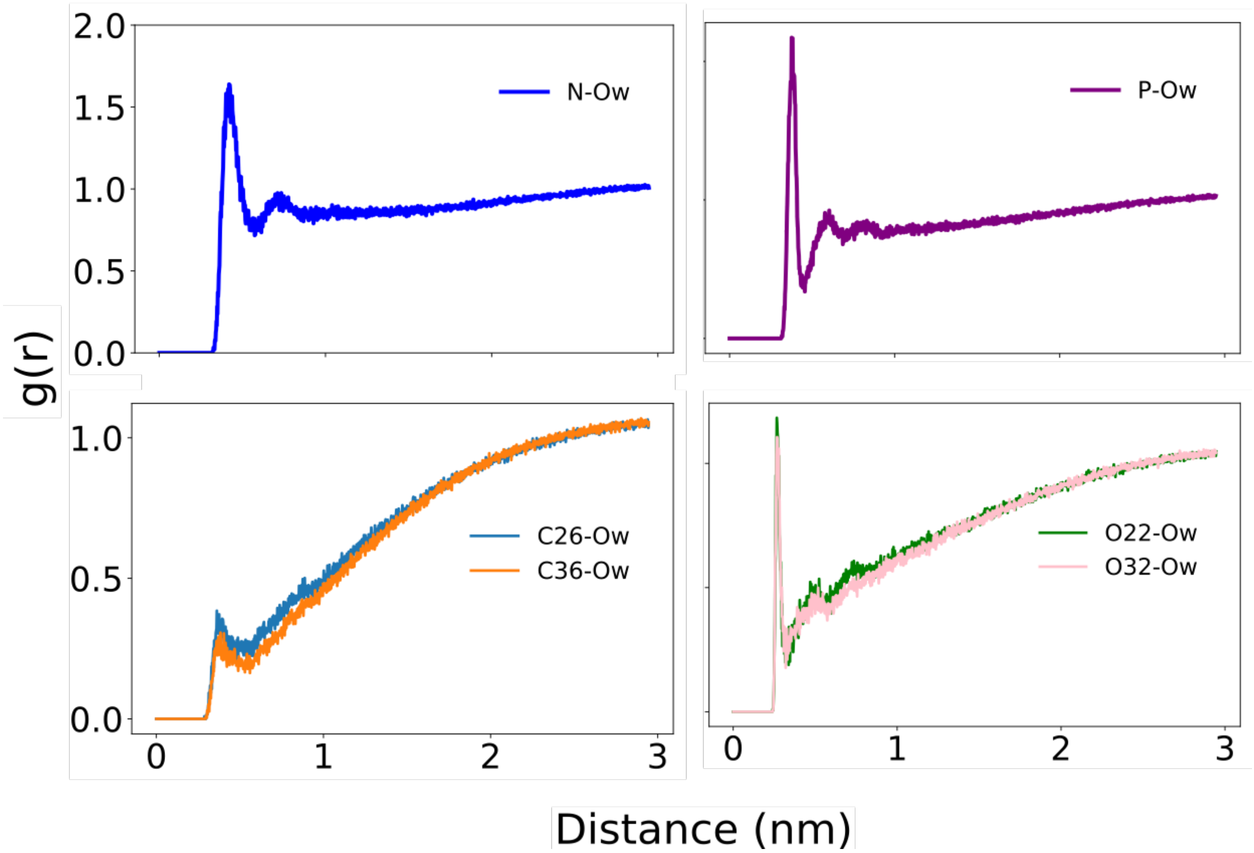

Figure S4: RDFs obtain for the C6 system containing 100% 2C6PC lipids.

Small angle neutron scattering model and measurements

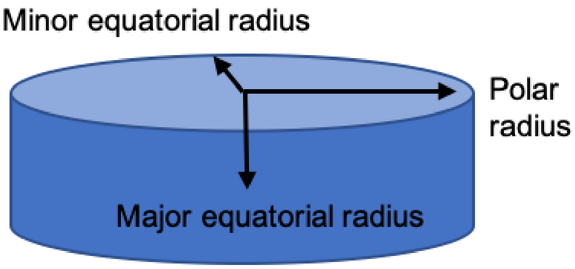

Figure S5: Illustration of a triaxial axial model used to plot the static and stopped-flow SANS plots shown in Figures 1 and 2

Several models were fitted to the static SANS measurements in order to determine the best fit for the 2C6PC micelle shape. Analysis of the fits as well as comparisons of aggregation numbers and hydration values (as seen in table 1) revealed that the 2C6PC micelles adopt a triaxial shape. The triaxial shape and the primary dimensions are illustrated in Figure S5. Conformation that the concentration of 2C6PC in the solution has negligible impact on shape and size of the resulting micelles can be see in Figure. S7, which shows little difference in the SANS profiles of 30 mM 2C6PC, 75 mM 2C6PC and 150 mM 2C6PC following scaling of each concentration to 30 mM and further adjustments to ensure a common background. This suggests that the same model can be applied to all 3 concentrations

In addition to the experiments described in the main text, the SANS profiles of 2C6PC in 3 different aqueous solvents were examined to understand the impact that different solvents and could have on the resulting 2C6PC micelle. Figure S6 demonstrates that there is no difference in the 2C6PC scattering profiles of the 3 solutions, suggesting that the 3 aqueous solvents used here do not alter the structure of the 2C6PC micelles.

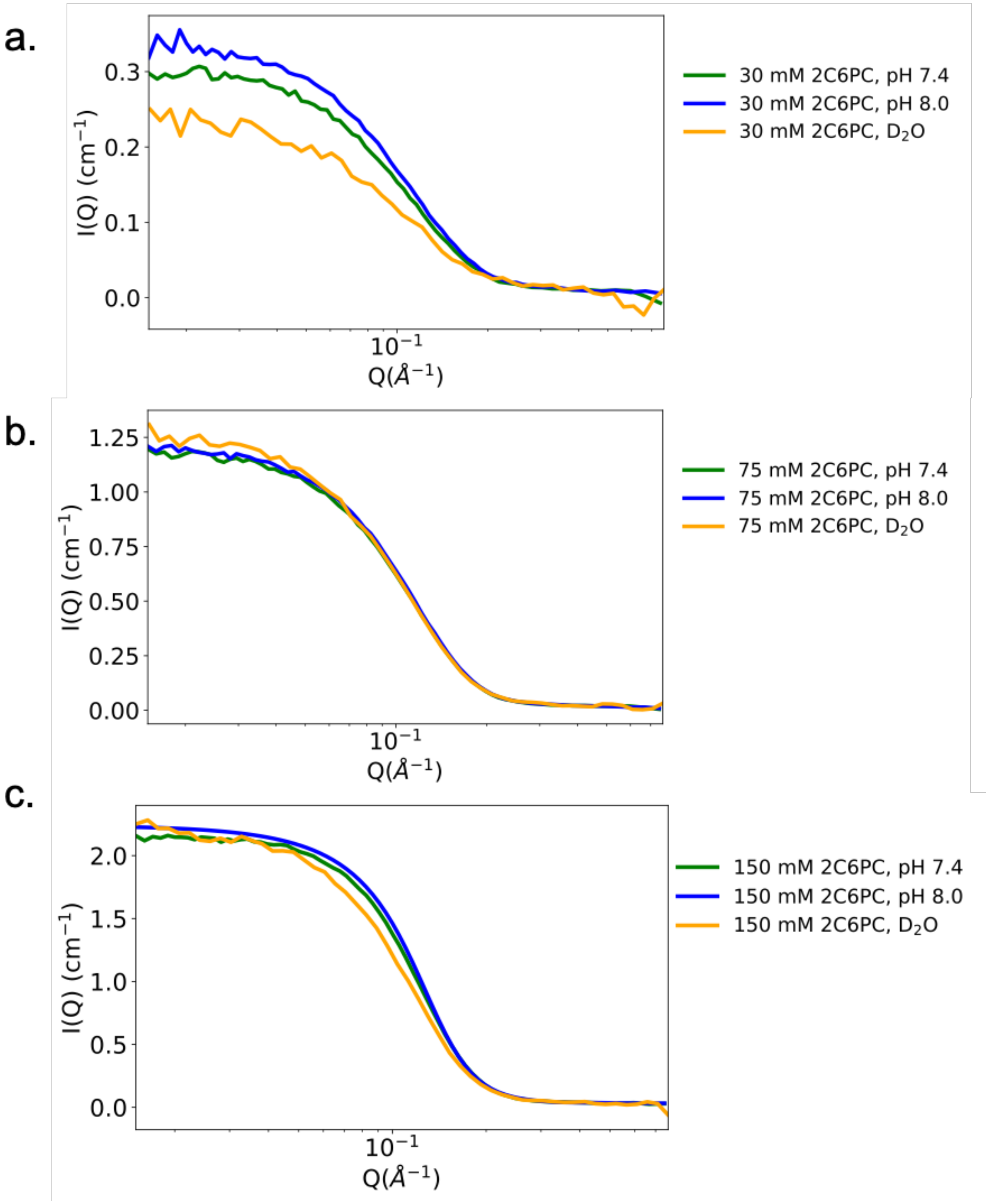

Figure S6: SANS profiles of a. 30 mM, b. 75 mM and c. 150 mM 2C6PC in 3 different aqueous solvents: D_2O, D_2O containing TRIS buffer, 150 mM NaCl and 5 mM $CaCl_2$ at pH 7.8 and D_2O containing TRIS buffer, 150 mM NaCl and 5 mM $CaCl_2$ at pH 8.0.

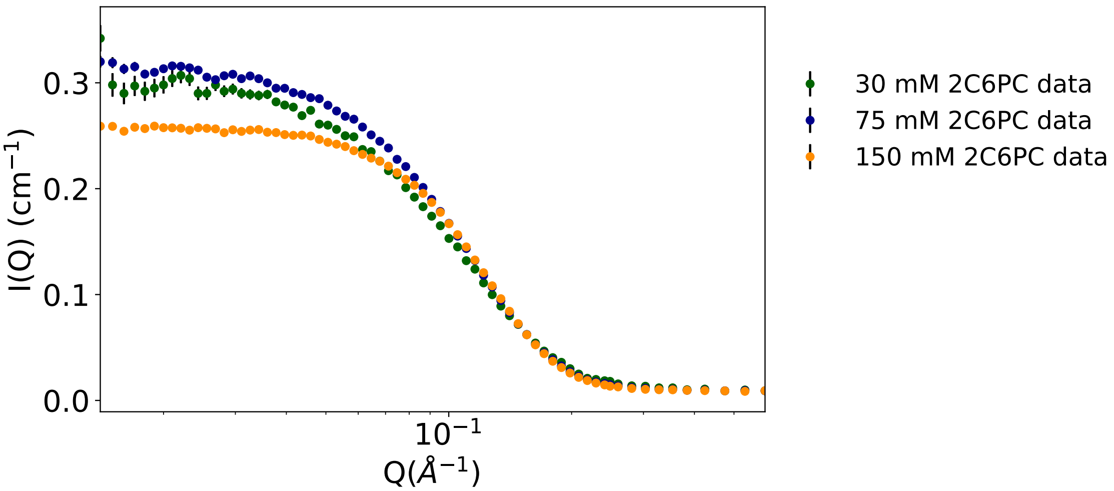

Figure S7: SANS profiles of the 30 mM, 75 mM and 150 mM 2C6PC at pH 7.4. The intensities of the 75 and 150 mM SANS experiments were scaled to 30 mM to show the overlap between all three profiles.
